# Psychosocial Reactions to Relocation to Nursing Homes in Chinese Older Adults

**DOI:** 10.1093/geroni/igaa057.1240

**Published:** 2020-12-16

**Authors:** Xiuyan Lan, Huimin Xiao, Ying Chen

**Affiliations:** 1 Fujian Provincial Hospital, Fuzhou, China; 2 Fujian Medical University, Fuzhou, China

## Abstract

This study aimed to elicit psychosocial reactions to relocation to nursing homes from older adults’ perspectives with a qualitative interview design. Narratives from 23 Chinese nursing home residents from Fuzhou, China in a life review program were recorded, transcribed into sentences, and analyzed with the qualitative content analysis. It revealed five stages of psychosocial reactions to relocation to nursing homes as fear, struggle, compromise, acceptance, and contribution. The first stage resulted from negative labels attached to nursing homes, disconnection to the society, difficulties in establishing new relationships, and being abandoned by their families. The second stage described the behaviors of struggle: complain about family members, think of going back home, pray to have a change, and take action to leave. The third stage described the keys to compromise: choices between maintaining the harmony in family relation and companionship of relatives, choices between professional care and family care, and choices between costs and effects of family care and nursing home care. The fourth stage described how they accept nursing home life: accept the life and yet with worries, affirm benefits of living in nursing homes, and embrace the nursing home life. The last stage resulted from sense of ownership and giving full play to self-worth. This study generated new insights into the knowledge on psychosocial reactions to relocation to nursing homes and provided both family members and nursing home staff with a direction for how to promote a smoother relocation process.

